# Effect of Network-Based Positive Psychological Nursing Model Combined With Elemene Injection on Negative Emotions, Immune Function and Quality of Life in Lung Cancer Patients Undergoing Chemotherapy in the Era of Big Data

**DOI:** 10.3389/fpubh.2022.897535

**Published:** 2022-05-06

**Authors:** Shilu Yang, Lijuan Zheng, Yan Sun, Zhuoyun Li

**Affiliations:** ^1^Intensive Care Unit, Wuhan Jinyintan Hospital, Wuhan, China; ^2^Department of Drug Resistant Tuberculosis, Wuhan Jinyintan Hospital, Wuhan, China; ^3^Jimo Nanquan Health Center, Qingdao, China; ^4^Department of Pharmacy, Qingdao Municipal Hospital, Qingdao, China

**Keywords:** network, positive psychology, elemene injection, lung cancer chemotherapy, negative emotions, immune function, quality of life (QOL)

## Abstract

**Background:**

With the development of big data, big data interpenetrate in every person's life. Health care is no exception to this trend, especially in regard to nursing analytics. The data that is analyzed and leveraged in this field is gathered from a variety of sources, including electronic health records (EHRs), medical histories, provider notes and mobile applications, creating an accumulation of personalized health information around each individual.

**Objective:**

To explore the effect of the network-based positive psychological nursing model combined with elemene injection on negative emotions, immune function and quality of life (QOL) in patients with lung cancer (LC) undergoing chemotherapy.

**Methods:**

The clinical data of 90 LC patients who underwent chemotherapy in our hospital from December 2020 to December 2021 were retrospectively analyzed, and the patients were equally split into experimental group (EG) and control group (CG) according to the order of enrollment. The patients in CG received routine nursing intervention during chemotherapy, while those in EG received the network-based positive psychological nursing model combined with elemene injection to compare negative emotions, immune function and quality of life (QOL) between the two groups.

**Results:**

Compared with CG, EG had notably higher immune function indexes (*P* < 0.001), lower serum VEGF and MMP-2 levels (*P* < 0.001), lower HAMA and HAMD scores (*P* < 0.05), and higher scores of PTGI, physiological function, psychological function, social function and physical function (*P* < 0.001).

**Conclusion:**

The network-based positive psychological nursing model combined with elemene injection is a reliable method to enhance the immune function and QOL of LC patients undergoing chemotherapy and alleviate their negative emotions, which has a high clinical application value.

## Introduction

Lung cancer (LC), mainly occurring in bronchial epithelial cells, is a common clinical malignant tumor with a high incidence (1) and seriously threatens human health. Thoracoscopic radical resection of lung cancer is a common surgical approach to treat LC (2), and postoperative chemotherapy is an indispensable adjuvant therapy. However, chemotherapy can trigger serious adverse reactions in patients, such as gastrointestinal reactions, vomiting and diarrhea (3, 4). In addition, patients during the period of chemotherapy often experience negative emotions due to worries about postoperative recovery and fear of tumors. In addition, the prevalence of the concept of cancer fear and the harm of LC itself expose patients to great psychological pressure, such as anxiety and tension. Besides, studies have found that immune function plays an important role in the disease development of malignant tumor patients, and cancer cells seriously affect the immune function of patients after the occurrence of tumors. At the same time, the toxic reactions of chemotherapy drugs also damage the normal physiological function of patients and result in a series of severe stress reactions, further damaging the immune system of patients, resulting in the decline of immune function and seriously affecting the quality of life (QOL). Therefore, how to alleviate the negative emotions of LC patients undergoing chemotherapy, enhance the immune function and QOL, and prevent disease recurrence is an urgent problem to be solved in clinic (5, 6). Routine nursing measures are carried out from health education, cognitive management and emotional support, which can hardly stimulate the potential of patients and leaves an unsatisfactory effect. Based on the theory of positive psychology, positive psychological intervention taps individual positive factors and stimulates the intrinsic potential of patients through their positive emotional experience and effective social support (7, 8). The development of Internet technology effectively solves the current lack of nursing resources in China. A report (9) shows that the network-based positive psychological nursing model enables patients to understand their diseases and maximizes the effect of psychological intervention through the instant push function of mobile phone APPs. A current study (10) has found that elemene injection can reduce the mitotic ability of tumor cells and induce their apoptosis, thus inhibiting their growth and proliferation, changing their immunogenicity, and strengthening the immune response of the body. Chemotherapy causes great physical and psychological pain to LC patients. In order to alleviate the pain of such patients, this study can play a guiding role in the clinical treatment of lung cancer, effectively improve the negative emotions of patients undergoing chemotherapy, reduce the economic burden of their families, and provide theoretical support for further research in the future. At present, few studies have confirmed the intervention effect of the network-based positive psychological nursing model combined with elemene injection on LC patients undergoing chemotherapy. In order to fill this gap and provide evidence-based evidence for more patients, this study is reported as follows.

## Materials and Methods

### General Information

The study subjects were 90 LC patients who received chemotherapy in our hospital from December 2020 to December 2021, and were equally split into experimental group (EG) and control group (CG) according to the order of enrollment. This study was approved by the ethics committee of *Jimo Nanquan Health Center*, following the Declaration of Helsinki (2013) (11).

### Inclusion and Exclusion Criteria

#### Inclusion Criteria

(1) Patients meeting the diagnostic criteria of LC and receiving chemotherapy; (2) patients with no history of epilepsy or cognitive impairment; and (3) patients with the stable condition and the approval of the attending doctor.

#### Exclusion Criteria

(1) Patents with a deteriorating condition and unstable vital signs; (2) patients with inability to understand the purpose of the researchers and the content of the questionnaire, and with severe cognitive impairment; and (3) patients who were allergic to the research drugs.

### Methods

Patients in CG received routine nursing intervention during chemotherapy through health education and professional guidance, including the pathogenesis, clinical manifestations and stages of disease development, precautions during chemotherapy, side effects of chemotherapy drugs, and dietary guidance (12, 13).

Patients in EG received the network-based positive psychological nursing model combined with elemene injection. The network-based positive psychological nursing model was carried out as below. (1) A network-based positive psychological nursing team was established, with 1 attending physician, 1 head nurse, 3 whole-course nurses, and 2 assistant nurses, aiming to organize the training on related knowledge of LC, chemotherapy and positive psychology, and carry out the assessment. The nurses could take the post only when they passed the assessment. (2) Register on the mobile APP. The LC health management platform was established, including modules such as chemotherapy period, psychological communication and patient assistance. The patients were guided to enter the platform and each module. The head nurse was the platform administrator and the whole-course nurses were responsible for the specific operations. (3) Online knowledge learning. According to the relevant content of positive psychology, combined with the psychological problems of LC patients undergoing chemotherapy, the head nurse organized and compiled the network courses of positive psychological intervention. The whole-course nurses pushed the contents on the WeChat public account weekly to guide patients to learn and use positive psychology to face the discomfort after chemotherapy. (4) Sharing of positive emotions on the platform. The patients were encouraged to write gratitude logs and upload them to the platform to cultivate their positive attitude. (5) Online mindfulness decompression training. The whole-course nurses led the patients to carry out online mindfulness decompression training, and guided them to exchange their feelings and ideas and express their psychological pain. Elemene injection (manufacturer: CSPC Pharmaceutical Group Co., Ltd.; NMPA approval No.: H20110114; specification: 10 ml: 0.2 g × 5 vials/box) was injected into patients, 400 mg/time, 1 time/d. With 21 days as a course of treatment, the patients received the continuous treatment of three courses. The dosage was appropriately adjusted according to the actual situation of patients during the treatment process.

### Observation Indexes

The fasting elbow venous blood of both groups after treatment was collected and centrifuged to prepare serum samples. The serum matrix metalloproteinases (MMP-2) and vascular endothelial growth factor (VEGF) were measured by ELISA. A protein immunoassay analyzer (model: CS-AutoBlot 48; manufacturer: Hunan Zhongrui Huxin Medical Technology Co., Ltd.) was used to detect the levels of immunoglobulin (Ig) G, IgA and IgM.

Hamilton Anxiety Scale (HAMA) (14) and Hamilton Depression Scale (HAMD) (15) were used to evaluate the negative emotions of both groups. The total score was 56 in HAMA and 68 in HAMD. Higher scores suggested worse emotions of patients.

#### Post-traumatic Growth Level

The Chinese version of Post-traumatic Growth Inventory (PTGI) (16) was applied to evaluate the post-traumatic growth level of patients, including life perception (4 items), personal strength (4 items), new possibility (3 items), relationship with others (6 items) and self-transformation (3 items). The Likert 6-level scoring method was adopted with 0–5 points from “no” to “very much”, and a total score of 100 points. A higher score indicated a higher post-traumatic growth level.

#### Quality of Life (QOL)

The Quality of Life (QOL) scale was used to evaluate the patients from psychological function, social function, physical function and physiological function, with each scoring 100 points. A higher score denoted better QOL.

### Statistical Methods

The data were processed by the professional statistical software SPSS26.0 and graphed by GraphPad Prism 7 (GraphPad Software, San Diego, USA). Enumeration data were tested by *X*^2^ and expressed as [*n* (%)], while measurement data were tested by *t*-test and expressed as Mean ± SD. When *P* < 0.05, the differences were statistically significant.

## Results

### Clinical Data

No obvious differences in sex ratio, times of chemotherapy, histological types and residence were observed between the two groups (*P* > 0.05) (see Table 1).

**Table 1:** Comparison of clinical data.

**Items**	**EG**	**CG**	***X*^2^/*t***	***P***
Gender			0.047	0.829
Male/Female	28/17	27/18		
Average age (Mean ± SD, yrs)	55.67 ± 4.65	55.24 ± 5.00	0.422	0.674
BMI (Mean ± SD, kg/m^2^)	20.64 ± 1.72	20.52 ± 1.56	0.347	0.730
**Times of chemotherapy**
The first time	13 (28.89)	14 (31.11)	0.053	0.818
The second time	14 (31.11)	15 (33.33)	0.051	0.822
The third time	12 (26.67)	11 (24.44)	0.058	0.809
Fourth or above	6 (13.33)	5 (11.11)	0.104	0.748
**Histological types**
Squamous cell carcinoma	22 (48.89)	21 (46.67)	0.045	0.833
Adenocarcinoma	14 (31.11)	17 (37.78)	0.443	0.506
Small cell carcinoma	6 (13.33)	5 (11.11)	0.104	0.748
Large cell carcinoma	3 (6.67)	2 (4.44)	0.212	0.645
Family income			0.179	0.673
>3,000 Yuan/(month·person)	25 (55.56)	23 (51.11)		
≤ 3,000 Yuan/ (month·person)	20 (44.44)	22 (48.89)		
Residence [*n* (%)]			0.403	0.525
Urban area	22 (48.89)	19 (42.22)		
Rural area	23 (51.11)	26 (57.78)		
**Education [*****n*** **(%)]**
College degree and above	4 (8.89)	6 (13.33)	0.450	0.502
High school	13 (28.89)	15 (33.33)	0.207	0.649
Junior high school and below	28 (62.22)	24 (53.33)	0.729	0.393

### Immune Function Indexes

All immune function indexes were notably higher in EG than in CG (*P* < 0.001), as presented in Table 2.

**Table 2:** Comparison of immune function indexes [Mean ± SD].

**Group**	***n***	**IgG**	**IgA**	**IgM**
EG	45	8.96 ± 0.95	6.69 ± 0.58	4.56 ± 0.25
CG	45	6.02 ± 0.49	3.15 ± 0.51	1.32 ± 0.11
*t*		18.450	30.747	79.576
*P*		<0.001	<0.001	<0.001

### Negative Emotions

The HAMA and HAMD scores in EG were remarkably lower compared with CG (*P* < 0.05) (see Table 3).

**Table 3:** Comparison of negative emotion scores [Mean ± SD].

**Group**	***n***	**HAMA score**	**HAMD score**
EG	45	15.33 ± 4.28	19.33 ± 5.96
CG	45	19.36 ± 5.27	23.82 ± 4.51
*t*		3.982	4.030
*P*		<0.05	<0.05

### Serological Indicators

**Figure 1:**
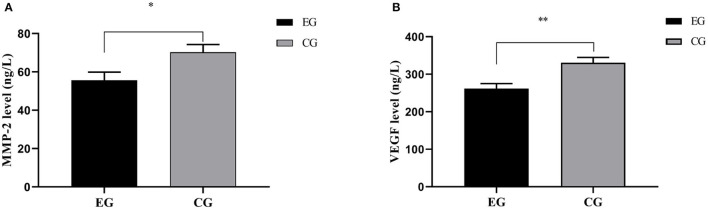
Comparison of serological indicators [Mean ± SD]. **(A)** Comparison of MMP-2 levels. The abscissa represented EG and CG, and the ordinate represented the MMP-2 level (ng/L). The MMP-2 levels of EG and CG were (55.61 ± 4.28) ng/L and (70.27 ± 4.06) ng/L, respectively. ^*^Indicated an obvious difference in the MMP-2 levels between the two groups (*t* = 16.670, *P* < 0.001). **(B)** Comparison of VEGF levels. The abscissa represented EG and CG, and the ordinate represented the VEGF level (ng/L). The VEGF levels of EG and CG were (261.86 ± 13.29) ng/L and (330.83 ± 14.09) ng/L, respectively. ^**^Indicated an obvious difference in the VEGF levels between the two groups (*t* = 23.887, *P* < 0.001).

The VEGF and MMP-2 levels were markedly lower in EG than in CG (*P* < 0.001) (Figure 1).

### Post-traumatic Growth Levels

**Figure 2:**
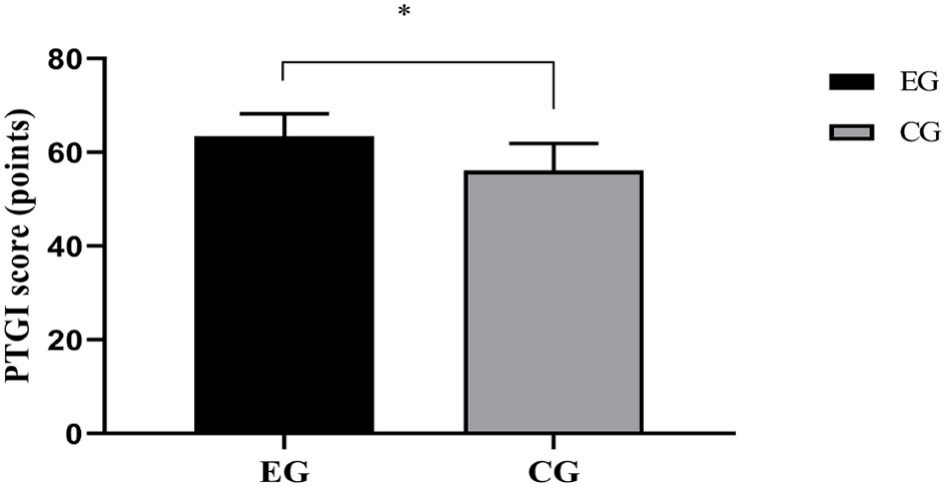
Comparison of PTGI scores [Mean ± SD]. The abscissa represented EG and CG, and the ordinate represented the PTGI score (points). The PTGI scores of EG and CG were (63.42 ± 4.78) and (56.18 ± 5.69), respectively. *indicated a notable difference in the PTGI scores between the two groups (*t* = 6.536, *P* < 0.001).

The PTGI score in EG was notably higher when compared with CG (*P* < 0.001), as shown in Figure 2.

### QOL

The scores of physiological function, psychological function, social function and physical function in EG were obviously higher compared with CG (*P* < 0.001), as shown in Table 4.

**Table 4:** Comparison of QOL scores [Mean ± SD].

**Group**	***n***	**Physiological function**	**Psychological function**	**Social function**	**Physical function**
EG	45	71.96 ± 3.75	75.44 ± 4.27	76.80 ± 4.64	77.60 ± 4.82
CG	45	67.91 ± 3.36	64.93 ± 3.14	67.82 ± 3.89	70.18 ± 4.74
*t*		5.396	13.302	9.949	7.363
*P*		<0.001	<0.001	<0.001	<0.001

## Discussion

Lung cancer (LC), the most common cause of cancer death, is also an important contributor to the damage and great economic pressure (17). Though playing an important role in the clinical treatment of LC, radiotherapy and chemotherapy trigger serious side effects, such as symptoms such as fatigue, cough, shortness of breath, and decreased appetite after chemotherapy, resulting in a serious decline in QOL of patients (18). Therefore, how to reduce or alleviate the side effects of chemotherapy and enhance the QOL of patients has become an urgent problem to be solved. With the insufficient understanding of the disease, LC patients are often accompanied by serious negative emotions such as annoyance, fear and depression with the clinical manifestations of distress, reduced self-esteem and crying once they are informed of the disease (19). While seriously disturbing the clinical treatment, these negative emotions also affect the sleep and QOL of patients. Supported by the Internet and based on the positive psychology, the network-based positive psychological nursing mode aims to alleviate the negative emotions of patients undergoing chemotherapy through the implementation of the positive psychological intervention, so as to explore their advantages, help them establish the confidence and courage to actively face the disease, alleviate negative emotions, and boost the QOL. A recent study (20) has confirmed that cognitive-behavioral therapy in network groups can effectively improve the anxiety and depression of LC patients after chemotherapy, and help patients to face treatment with a positive attitude. However, this study only discusses the psychological emotions of patients, and lacks in-depth discussions on their immune function and QOL. Elemene injection is an anti-cancer drug developed independently in China. With certain curative effects on a variety of malignant tumors, it can enhance the immune function of patients, and interfere with the synthesis of DNA and RNA in tumor cells accompanied by less effects on normal cells, which has been confirmed in tumor diseases such as malignant pleural effusion, advanced gastric cancer and esophageal cancer (21, 22). This paper discussed the effect of the network-based positive psychological nursing model combined with elemene injection on LC patients undergoing chemotherapy, and analyzed the immune indexes and serological indexes of patients, so as to clarify the clinical application value. Based on the above method, this experiment selected 90 LC patients treated with chemotherapy in our hospital as the research subjects, in which the EG group received the network-based positive psychological nursing model combined with elemene injection to analyze the effect of different treatment methods on the patients and clarify the clinical application value by comparing the immune function indexes, serological indicators and other indexes after treatment between the two groups. As shown in Table 1, no obvious differences in age, BMI, times of chemotherapy and histological types were observed between the two groups (*P* > 0.05), which met the requirements of clinical trials.

Under pathological conditions, activated MMP-2 can degrade the extracellular matrix components and break through the matrix barrier, leading to tumor invasion and metastasis. VEGF is a highly specific angiogenic growth factor to promote the formation of new blood vessels, which maintains a high level in many tumor diseases (23). This study showed that the VEGF and MMP-2 levels after treatment were obviously lower in EG than in CG (*P* < 0.001), suggesting that this therapeutic schedule can significantly reduce the VEGF and MMP-2 levels in LC patients undergoing chemotherapy, possibly because elemene injection with the effect of anti-angiogenesis can effectively inhibit the proliferation, invasion and migration of tumor cells (24). In terms of the improvement of negative emotions, the HAMA and HAMD scores in EG were markedly lower compared with CG (*P* < 0.05). This is because the network-based positive psychological nursing model takes individual negative psychological level as the research goal and adopts positive emotions to resist negative emotions, so as to stimulate personal potential, tap personal advantages, help patients actively face the setbacks and misfortunes, and urge them to experience the joy and success experience brought by positive actions. Therefore, their self-worth and sense of identity are enhanced to fundamentally improve the negative emotions. As for immune function and QOL, they were notably better in EG than in CG after intervention (*P* < 0.001). The reason may be that elemene injection is metabolized through the lungs and has a high concentration in lung tissues with less side effects and good tolerance in patients, which can effectively prolong the survival time and enhance the QOL of patients. These effects of elemene injection have been confirmed in the third-line treatment of advanced non-small-cell lung cancer (25). In addition, an animal experiment (26) has shown that elemene injection can improve the T lymphocyte functions of mice, and enhance the antigen presentation and expression of immune-related factors. The network-based positive psychological nursing model pushes network courses about the positive psychological intervention to patients via mobile APPs, and encourages them to write gratitude logs to imperceptibly cultivate their optimistic attitude toward life, transfer the pain caused by adverse reactions of chemotherapy, and boost the QOL. Contributions: After the network-based positive psychological nursing model combined with elemene injection was applied to LC patients undergoing chemotherapy, this study was aimed to effectively alleviate the negative emotions of such patients, reduce the impact of chemotherapy on their immune function, and enhance QOL by evaluating the clinical indicators of patients after treatment. This nursing model also provides an important basis for improving the treatment confidence osf cancer patients, reflects great progress and development of clinical nursing and has comprehensive guiding significance in modern health education and clinical practice, which will undoubtedly become the future direction of medicine. However, psychological nursing based on positive psychology has not yet formed a unified operation mode, which needs to be continuously expanded and deepened. In addition, its localization research under the cultural background in China needs to be further strengthened.

## Conclusion

In conclusion, the network-based positive psychological nursing model combined with elemene injection is a reliable method to enhance the immune function and QOL of LC patients undergoing chemotherapy. This intervention is beneficial to patients and is superior to routine nursing intervention in improving patients' negative emotions, reducing their serological indicators and enhancing their QOL, with significant effects.

## Data Availability Statement

The original contributions presented in the study are included in the article/supplementary material, further inquiries can be directed to the corresponding author.

## Ethics Statement

The studies involving human participants were reviewed and approved by the Ethics Committee of Jimo Nanquan Health Center. The patients/participants provided their written informed consent to participate in this study. Written informed consent was obtained from the individual(s) for the publication of any potentially identifiable images or data included in this article.

## Author Contributions

SY, LZ, and ZL: conception and design, data analysis, and interpretation. All authors contributed in administrative support, provision of study materials or patients, collection, assembly of data, manuscript writing, and final approval of manuscript.

## Conflict of Interest

The authors declare that the research was conducted in the absence of any commercial or financial relationships that could be construed as a potential conflict of interest.

## Publisher's Note

All claims expressed in this article are solely those of the authors and do not necessarily represent those of their affiliated organizations, or those of the publisher, the editors and the reviewers. Any product that may be evaluated in this article, or claim that may be made by its manufacturer, is not guaranteed or endorsed by the publisher.
